# Applying Nightingale charts to evaluate the heterogeneity of biomedical waste in a Hospital

**DOI:** 10.1590/0104-1169.3309.2499

**Published:** 2014

**Authors:** Janini Cristina Paiz, Marcio Bigolin, Vania Elisabete Schneider, Nilva Lúcia Rech Stedile

**Affiliations:** 1 RN; 2 Master's student, Universidade Federal do Rio Grande do Sul, Porto Alegre, RS, Brazil; 3 PhD, Professor, Universidade de Caxias do Sul, Caxias do Sul, RS, Brazil; 4 PhD, Professor, Universidade de Caxias do Sul, Caxias do Sul, RS, Brazil. Post-doctoral fellow, Fundação Oswaldo Cruz, Rio de Janeiro, RJ, Brazil

**Keywords:** Waste Management, Medical Waste, Occupational Risks

## Abstract

**OBJECTIVES:**

to evaluate the heterogeneity of biomedical waste (BW) using Nightingale charts.

**METHOD:**

cross-sectional study consisting of data collection on wastes (direct observation of receptacles, physical characterisation, and gravimetric composition), development of a Management Information System, and creation of statistical charts.

**RESULTS:**

the wastes with the greatest degree of heterogeneity are, in order, recyclable, infectious, and organic wastes; chemical waste had the most efficient segregation; Nightingale charts are useful for quick visualisation and systematisation of information on heterogeneity.

**CONCLUSION:**

the development of a management information system and the use of Nightingale charts allows for the identification and correction of errors in waste segregation, which increase health risks and contamination by infectious and chemical wastes and reduce the sale and profit from recyclables.

## Introduction

Biomedical waste (BW) is defined as waste related to human or animal health services, including home care and fieldwork services; analytical laboratories for health products; morgues, funeral homes, and embalming services; drugstores and pharmacies; educational and health research facilities; zoonosis control centres; mobile health units; and acupuncture services^(^ 1 ^-^ 2 ^)^. According to the resolutions adopted in Brazil,^(^ 1 ^-^ 2 ^)^ BW is divided into five groups: Group A (infectious), Group B (chemical), Group C (radioactive), Group D (common), and Group E (piercing and cutting).

When BW is produced, the professionals who handle it are solely responsible for segregating it according to its characteristics and disposing of it in adequate receptacles. Segregation is the key step in the management of BW; inadequacies here compromise all other steps.

Several factors can contribute to errors in segregation. Among them is the lack of specific knowledge on BW by the professionals who generate and manipulate it and the minimal importance normally given to BW by these same professionals compared to other tasks they perform. There are also behaviour patterns derived from the similarity between household waste and biomedical waste that lead individuals (including health professionals) to dispose of BW generated at home together with common waste. Common examples are diabetic patients - who take injectable insulin daily - and injectable drug users, who generate piercing and cutting waste that is usually disposed of together with common household waste.

Household wastes and wastes inadequately disposed of in health facilities due to the poor management of BW in Brazil lead to several problems that can affect the health of the population and the health of workers who have direct contact with these wastes^(^ 3 ^)^. BW constitutes a favourable environment for many organisms, which can become vectors and reservoirs of various pathologies capable of transmission by rodents, insects, and other animals. This fact makes special handling of BW mandatory, including specific types of treatment and disposal.

As early as 1978, studies confirmed the presence of pathogenic microorganisms in BW. The most common are enteric gram-negative bacilli (coliforms, *Salmonella typhi*, *Shigella sp. and Pseudomonas sp.*), gram-positive cocci (*Streptococcus and Staphylococcus aureus*), fungi (*Candida albicans*), and viruses (hepatitis A and B, enteric virus, and polio type 1)^(^ 4 ^)^.

Studies conducted to evaluate indicators of environmental contamination resulting from microorganisms found in BW show that contamination can occur through the air, water, and/or soil^(^ 5 ^)^. The microorganisms that show the highest individual risks (host penetration) via air contamination are *Mycobacterium tuberculosis* and *Staphylococcus aureus*. Water contamination incurs the risk of ingestion of contaminated water, and infection by the Hepatitis A virus and the bacterium* Escherichia coli *predominate in this medium. Albeit with lower frequency, Hepatitis B and *Clostridium perfringens* infections may also occur. Microorganisms with high capacity for soil contamination include *Pseudomonas aeruginosa*, the Hepatitis B virus, the *Enterococci, *and* Staphylococcus aureus* ^(^ 5 ^)^.

The presence of pathogenic microorganisms in BW reaffirms the need for the use of Personal Protective Equipment (PPE) by the professionals who handle it, as they may be victims of occupational accidents or become vectors of infection to patients. Thus, investments must be made to train workers involved with BW in the use of protective equipment and appropriate handling of this waste^(^ 6 ^)^.

The risks associated with improper handling of BW include inappropriate or even non-existent hazardous waste segregation and the mixing of such waste with common waste, which promotes contamination of the latter, increasing the amount of contaminated material and the related risks; inappropriate segregation of piercing and cutting waste without using mechanical protection, which is responsible for the largest number of occupational accidents in health facilities; and the disposal of BW in dumps, improper landfills, or together with household waste, which presents a serious risk of injury to waste collectors and of environmental contamination near the disposal site. This improper handling increases the risk of exposure, compromising the health of workers, patients, and the environment^(^ 7 ^)^.

**Figure 1:**
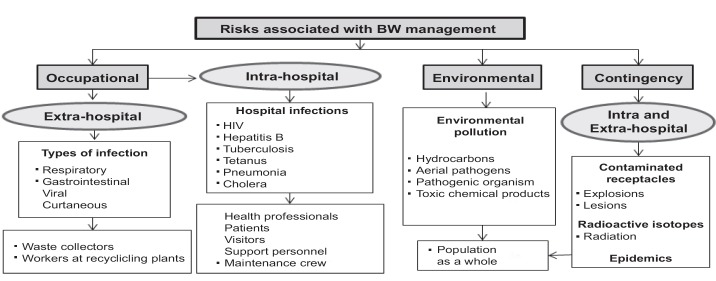
Risks associated with Biomedical Waste Management

In this context, the risks associated with BW management can be divided into three major areas: occupational, environmental, and contingency. Figure 1 presents the risks associated with the management process, showing the areas in which they occur, the type of risk, and which population is exposed to it.

Therefore, for safe management, it is essential that all individuals working in the health facility know the risks associated with their activities, have clear responsibilities, and are trained to perform the procedures related to waste management, as occupational accidents usually occur due to the combination of a number of factors or inadequacies^(^ 8 ^)^_._

This study was conducted in a service, teaching, and research hospital, in the Northeast region of Rio Grande do Sul, Brazil, which is a reference hospital in the region, providing services through the public Unified Health System. The guiding question was the following: can Nightingale charts assess the degree of heterogeneity of BW? 

The objectives of this study were to assess the BW management system with emphasis on segregation efficiency (heterogeneity), which is the critical step in the waste management process, and to assess whether a Management Information System (MIS) and Nightingale charts are useful for analysing the efficiency of the management of these wastes.

## Methods

This work is a cross-sectional study, the methods of which encompassed two distinct stages. The first stage consisted of data collection on the management of BW based on the direct observation, physical characterisation, and gravimetric composition of BW^(^ 9 ^)^. The second stage consisted of implementing and maintaining a Management Information System (MIS) to record and process waste characterisation data, followed by systematisation and presentation in a Coxcomb chart format^(^ 10 ^)^, allowing the historical retrieval of a tool for data presentation in the field of nursing.

 Data collection related to waste management was performed by: 

*- Direct observation of the study site:* performed before each characterisation, with the goal of identifying and evaluating the presence, quantity, and location of receptacles, as well as the adequacies and inadequacies of the management process (internal collection, internal disposal, internal transport, and external disposal).

*- Assessment of waste generation by different hospital divisions during a 24-hour period:* the wastes were classified according to their different Groups (A: infectious B: chemical, D: common and recyclable)^(^ 1 ^)^ as well as to their generating division and weight. This information enables the quantitative assessment of waste generation, providing indices and indicators of mean waste generation in each category. 

*- Qualitative evaluation of waste:* performed by characterising a sample unit of 200 L for the infectious, common, recyclable, and chemical categories, extracted from the amount generated during the 24 hour period. The characterisation consists of opening the receptacles that compose the sample, examining the contents, and segregating the contents properly, repeating the weighing process.

The information obtained from the characterisation enables the qualitative assessment of waste generation and the generation of performance indexes and indicators for each category. The wastes were previously identified with the division and the date of collection, allowing for estimation of the degree of heterogeneity by division and of the costs of treatment and disposal of different waste categories.

This qualitative and quantitative evaluation was performed over six consecutive months (February to August 2012), selecting a different day of the week every month, so that every day of the week was evaluated, allowing the identification of possible changes in weight generation (kg) and/or in heterogeneity. Because this work is a service evaluation study, it was approved by the local ethics committee (Comitê de Ética em Pesquisa - CEP/FUCS).

To manage the data resulting from this research and to create reports, charts, and forecasts that support decision-making, a Management Information System (MIS) is being implemented. This MIS has a life cycle and can be developed in an interactive and incremental way. The proposed model is known as spiral model: each turn of the spiral refines the problem and adds details to the requirements^(^ 11 ^)^.

The system is being guided by a variation of the Unified Process^(^ 12 ^)^. Data are accessed through a web interface developed in PHP (Hypertext Pre-Processor) and stored in a database implemented by the open source Data Base Management System (DBMS) PostgreSQL. DBMS are a collection of programs to enable users to maintain a database and thus facilitate the process of defining, building, manipulating, and sharing data^(^ 13 ^)^. 

The information management model uses multidimensional modelling, also known as *StarSchema*, which is widely used in data warehouses. This modelling consists of organising the information structures into facts and dimensions^(^ 13 ^)^. A fact contains useful measures of business processes (in this study, represented by weighing and characterisation), while a dimension represents the context (in this study the type of waste, generating division, and date). Because it is not normalised, this model is designed for rapid querying and cannot be used as the main form of data entry. 

To evaluate the results, diagrams presented in 1859 by Florence Nightingale were used, generated by the PROTOVIS^1*^ library. Through her work as a nurse in the Crimean War, Florence Nightingale was a pioneer in establishing the importance of sanitation in hospitals. She gathered data on the number of deaths related to sanitation, and because of her new methods of communicating these data, she was also a pioneer in applied statistics^(^ 10 ^)^. Nightingale used charts that were later called Coxcomb.

**Figure 2:**
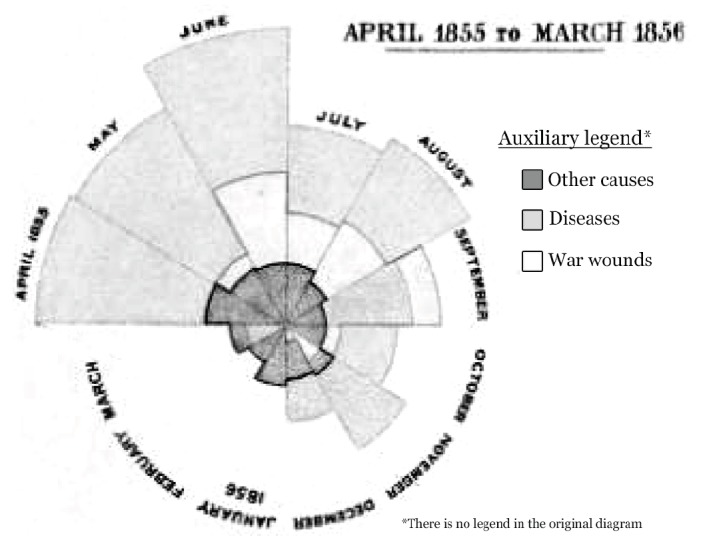
Original Coxcomb diagram(10)

The role of Florence Nightingale in the history of statistics is of interest for many reasons. Her role as a social activist and her visualisation of statistical data presented in charts and diagrams that could be used as powerful arguments for medical reform are of great importance. Social phenomena could be objectively measured and subjected to mathematical analysis. The Coxcomb chart was innovative in the collection, tabulation, interpretation, and graphical presentation of descriptive statistics. Figure 2 shows an adaptation of the original statistical chart (Coxcomb). This diagram shows the incidence and prevalence of death due to three main causes at the time: war wounds/injuries, diseases, or other causes. 

Even if this diagram is limited to the presentation of descriptive statistics, it facilitates the visualisation of temporal data and of the evolution of certain phenomena. In this study, it allows for visualisation of the degree of heterogeneity and of the evolution of the BW management process.

## Results

**Figure 3:**
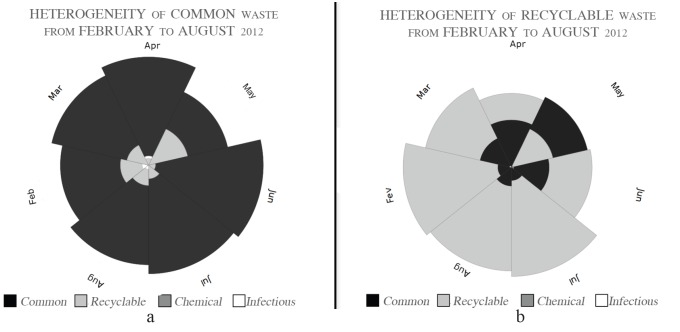
Heterogeneity of common and recyclable waste from February to August 2012

**Figure 4:**
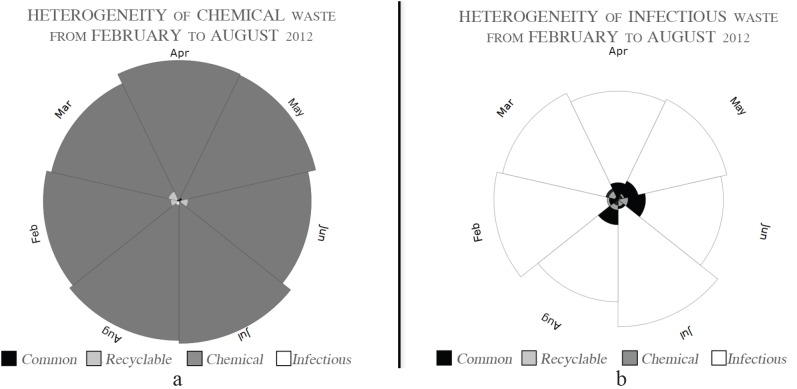
Heterogeneity of infectious and chemical waste from February to August 2012

Figures 3(a, b) and 4(a, b) show the heterogeneity of BW (common, recyclable, infectious and chemical) between February and August 2012 at the hospital studied.

Based on Figure 3a, it is observed that the periods in which common waste had the highest heterogeneity are the months of May and February. In this category, a marked amount of recyclables incorrectly segregated as common waste is observed. It is also observed in Figure 3a that in February, a mixture of infectious waste with common waste was found (although in small amount).

Figure 3b shows that recyclable waste has a high degree of heterogeneity, greater than observed in all other categories. The vast majority of waste found mixed with recyclables was common waste (organic).

Figure 4 shows the heterogeneity of chemical and infectious waste between the months of February and August 2012 at the hospital studied.

Based on Figure 4b, it is possible to observe that all categories of BW are mixed with infectious waste, especially common and recyclable waste, even if in small amounts. Figure 4a shows that the least mixed waste is chemical waste. The low heterogeneity of this category was observed in all samples. Even in small quantities, recyclable and/or common waste appears in every month assessed; however, the efficiency of the segregation of chemical waste is higher than 90%. Table 1 shows the mean heterogeneity and the standard deviation in the different waste categories evaluated from February to August 2012.

**Table 1:** Average heterogeneity and standard deviation of BW assessed between February and August 2012.

Waste	Heterogeneity	*x*	Σ	Waste	Heterogeneity		*x*		Σ
Recyclable	Common	28.10	18.96	Chemical		Common	0.72	0.80	
	Infectious	0.32	0.55			Infectious	0.63	0.91	
	Chemical	0.47	0.83			Chemical	94.94	3.25	
	Recyclable	70.95	19.50			Recyclable	2.92	3.28	
Common	Common	82.20	9.80	Infectious		Common	10.99	6.43	
	Infectious	1.51	2.14			Infectious	79.62	6.64	
	Chemical	0.12	0.34			Chemical	1.90	3.19	
	Recyclable	16.21	9.87			Recyclable	7.10	2.48	

Table 1 reveals that the wastes with the highest indices of mean heterogeneity are recyclable (70.95), infectious (79.62), and common (82.20) waste, whereas chemical waste has the best index of segregation (94.94). The wastes that showed the highest standard deviation, in order, were recyclable, common and infectious, while chemical waste had the lowest standard deviation, justifying the data presented in the chart.

## Discussion

Given that the facility studied is a teaching hospital with a high turnover of students, when the months corresponding to the beginning of the semester are compared with the months corresponding to the end of the semester, differences in heterogeneity are observed. These values are smaller, for example, in July for common and recyclable waste (Figures 3a and 3b), which is the month in which student turnover is also smallest.

The mixture shown in Figures 3a and 3b reflects an increased amount of organic waste and reduction in the reuse of recyclables, as the latter is not sent to sorting or recycling centres (where it creates jobs and income) but taken instead to landfills or dumps, losing its commercial value.

Although the mixture of infectious with common waste (Figure 3a) is very small, it contaminates the entire sample, as once infectious waste comes into contact with other waste, the entire mass becomes infectious and must be treated as such. This contamination results in increased environmental and health risks, both inside the hospital (health professionals, patients and cleaning crew) and outside the hospital (workers involved with external waste collection, treatment and final disposal)^(^ 7 ^)^. Studies show that infectious wastes, especially piercing and cutting wastes, are the main categories responsible for occupational accidents ^(^ 14 ^)^.

In May, more than 50% of the waste segregated as recyclable was, in fact, organic, which reduces the quality of recyclable waste destined for sorting centres, attracts vectors, and interferes in the work of waste collectors. It also increases the costs of recycling because it is necessary to re-categorize the waste for allocation to a landfill.

The presence of common and recyclable waste mixed with chemical and infectious waste (Figures 4a and 4b) increases the costs of the treatment of infectious waste and reduces the reuse of recyclables^(^ 15 ^)^, thus constituting a double loss. Chemical wastes, when segregated improperly, can result in human poisoning and injury in many ways. Injuries can be caused by contact with the product as well as by inhalation of gases or ingestion of contaminated food and water^(^ 16 ^)^. Despite the possibility of accidents involving chemical and infectious waste, piercing and cutting waste causes the highest number of occupational accidents^(^ 17 ^)^.

It is worth noting that receptacles for infectious waste harbour biological fluids from several patients, and an accident involving one of these materials requires the worker to undergo a mandatory prophylaxis due to lack of knowledge of the source-patient, exposing the worker to strong side effects of medications and procedures that would not otherwise be necessary^(^ 18 ^)^.

The characterisation of BW is an essential tool in the assessment of waste generated, and it allows for precise identification of the types of problems and the sites responsible. It also serves as a basis to evaluate risks with BW management and to generate indexes and indicators of efficiency and effectiveness, which are useful in improving the process. In this study, the indicators of segregation efficiency were 94.94% for chemical wastes, 82.20% for common waste (organic), 79.62% for infectious waste, and 70.95% for recyclable waste. These indicators show the need for improvement of the management system and continuing education programs, both at the undergraduate level and in the context of health care services. Continuing education is an essential tool for the maintenance of correct attitudes and adequate behaviours for the implementation of the Plan and also for the development of new behaviours, especially in a facility involved in the education and training of health professionals with a high turnover of students from different courses and training stages.

Reducing risks to health professionals and patients, as well as environmental risks due to the improper management of wastes from healthcare services, depends on a number of coordinated actions involving all parties responsible for health care.

The use of a MIS in health care facilities, aside from allowing the storage, access, and quick search of data, provides a temporal view of the phenomenon and tends to maintain the necessary behavioural changes to achieve 100% efficiency in the management process. In other words: it is crucial to obtain a zero index of heterogeneity.

The generation of indexes and indicators is essential for self-monitoring of the management system and allows evaluation of the system's behaviour over time, supporting intervention and optimisation of the performance of the waste management process and of the continuing education programs. It also allows comparative analysis with other institutions. In this sense, it is emphasized that these performance evaluation tools can assist health care facilities, providing an overview of the situation of the procedures and practices adopted^(19).^

Nightingale charts are also suitable for this purpose due to allowing rapid visualization of the statistical data and influencing the BW decision-making process and the consequent reduction of risks to physical, environmental, and organisational health. As they allow for temporal data assessment, these charts indicate the months in which there were changes in the degree of heterogeneity of the waste categories evaluated, which can be confirmed by analysis of the standard deviation.

## Conclusions

Data analysis allows the assessment of different aspects of the BW management system in this facility as follows: a) the wastes that showed greater heterogeneity in the months studied were recyclable (70.95), infectious (79.62), and common (82.2); b) the inadequacies and consequent heterogeneity are greater during the first months of the study, most likely related to the beginning of the rotation of new students from different hospital divisions areas; c) the greatest health risks are primarily related to inadequate segregation of infectious and chemical wastes, especially mixed with common and recyclable wastes, as the individuals who handle the waste do not expect to find infectious and chemical wastes and do not wear adequate protection; d) the presence of infectious and chemical wastes mixed with common and recyclable wastes increases treatment costs, as all wastes of mixed nature must be considered infectious or chemical, and when the mixture is not noticed, it compromises occupational and environmental health; e) the presence of common and recyclable wastes together with infectious and chemical wastes necessarily increases the costs of treatment, as they must be treated prior to final disposal; f) the system efficiency, shown by indices and indicators, also reveals opportunities for improvement and optimisation of the management system. 

Nightingale charts were useful in the analysis and processing of data related to BW because they allowed for rapid visualisation of the degree of heterogeneity and enabled the evaluation of temporal data and monitoring of the evolution of BW segregation, as well as identifying the months in which there are greater inadequacies and inferring possible causes. The proposed MIS is a useful tool not only in organising and recording data but also in maintaining a temporal data sequence, which allows more complex analysis and a greater understanding of the evolution of the phenomenon over time.

Finally, the importance of constant improvement of the management system, whether by the use of new technologies or by the continuing training of professionals involved in the problem, is emphasised.
